# Platelets in Inflammation: Regulation of Leukocyte Activities and Vascular Repair

**DOI:** 10.3389/fimmu.2014.00678

**Published:** 2015-01-06

**Authors:** Angèle Gros, Véronique Ollivier, Benoît Ho-Tin-Noé

**Affiliations:** ^1^Université Paris Diderot, Sorbonne Paris Cité, Paris, France; ^2^Unit 1148, Laboratory for Vascular Translational Science, INSERM, Paris, France

**Keywords:** neutrophils, platelets, inflammation, monocytes, vascular permeability, bleeding, thrombocytopenia, ITAM

## Abstract

There is now a large body of evidence that platelets are central actors of inflammatory reactions. Indeed, platelets play a significant role in a variety of inflammatory diseases. These diseases include conditions as varied as atherosclerosis, arthritis, dermatitis, glomerulonephritis, or acute lung injury. In this context, one can note that inflammation is a convenient but imprecise catch-all term that is used to cover a wide range of situations. Therefore, when discussing the role of platelets in inflammation, it is important to clearly define the pathophysiological context and the exact stage of the reaction. Inflammatory reactions are indeed multistep processes that can be either acute or chronic, and their sequence can vary greatly depending on the situation and organ concerned. Here, we focus on how platelets contribute to inflammatory reactions involving recruitment of neutrophils and/or macrophages. Specifically, we review past and recent data showing that platelets intervene at various stages of these reactions to regulate parameters such as endothelial permeability, the recruitment of neutrophils and macrophages and their effector functions, as well as inflammatory bleeding. The mechanisms underlying these various modulating effect of platelets are also discussed.

## Platelets Control Endothelial Permeability and Leukocyte Infiltration

While platelets have long been known to promote the semi-permeable barrier function of the resting endothelium (1), it has become equally clear that under inflammatory conditions, platelets can promote vascular permeability. Indeed, both edema formation and leukocyte infiltration have been shown to be markedly reduced by thrombocytopenia in multiple models of acute or chronic inflammation. For instance, immunodepletion of platelets was shown to inhibit neutrophil extravasation and the efflux of Evans blue from mesenteric venules in a mouse model of thioglycollate-induced peritonitis (2). Thrombocytopenia also resulted in reduced skin edema and infiltration of neutrophils, eosinophils, and mast cells in models of cutaneous leukocytoclastic vasculitis (3) and of chronic contact hypersensitivity (4). These pro-inflammatory effects of platelets were also reported in the inflamed lungs (5–7), the very same organ where the anti-permeability effect of platelets was demonstrated in unchallenged thrombocytopenic animals (8).

Notably, while in the above-cited examples the efflux of leukocytes and plasma were both promoted by platelets, situations where only one of these parameters was impacted by platelet depletion have also been reported. In a mouse model of irritant contact dermatitis, experimental thrombocytopenia led to reduced edema formation without affecting neutrophil infiltration (9). Platelet depletion also resulted in complete abrogation of protein leakage without affecting neutrophil recruitment to the lungs in a model of transfusion-related acute lung injury (7). Concordantly, thrombocytopenia fully prevented endothelial gap formation and vascular leakage during experimental arthritis but only partially reduced the overall arthritis severity (10). Therefore, although promotion of plasma and leukocyte extravasation by platelets may share common mechanistic aspects, they are clearly distinct parameters with their own distinct regulatory mechanisms. Interestingly, it was recently shown that leukocyte extravasation and vascular permeability can be controlled separately by different tyrosine residues of VE-cadherin (11). Whether such a mechanism contributes to differential regulation of vascular permeability and leukocyte infiltration by platelets remains to be demonstrated but these results suggest that distinct and selective regulation of these two parameters is possible at the endothelial junction level.

Recruitment of platelets to the inflamed vasculature and local release of soluble compounds from activated platelets likely constitute the predominant basic mechanisms common to regulation of both vascular permeability and leukocyte extravasation by platelets. Interactions between platelets and inflamed vessels have been shown to be an early event in various models of acute and chronic inflammation (7, 9, 10, 12–19). Various adhesion receptors can support the early recruitment of platelets to inflamed microvessels and the subsequent actions of platelets on vascular permeability and leukocyte extravasation. The nature of these receptors is highly dependent on the inflammatory context. While platelet P-selectin was crucial for platelet recruitment and leukocyte extravasation in experimental colitis (20), acute lung injury (5), chronic cutaneous hypersensitivity (4), allergic asthma (21), glomerulonephritis (17), and cerebral and intestinal ischemia/reperfusion (13, 14), it played only a minor role in the recruitment of neutrophils in thioglycollate-induced peritonitis (2). Likewise, GPIb was critical for platelet recruitment and leukocyte invasion at atheromatous lesion-prone sites in hypercholesterolemic mice (12) and during peritonitis (2) but dispensable for early recruitment of platelets to the inflamed glomerulus (19). In addition to P-selectin and GPIb, GPVI, GPIIb–IIIa, CD40L are among the other platelet receptors that have been reported to be involved in platelet recruitment and action on vascular permeability and/or leukocyte recruitment (Table 1).

**Table 1:** **Regulation of vascular permeability and leukocyte recruitment by platelets**.

	Inflammatory model	Receptors and/or soluble factors involved	References
Vascular permeability	Arthritis	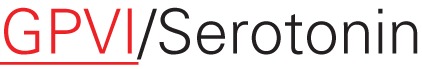	Cloutier et al. (10)
	Peritonitis	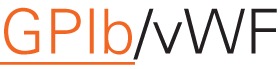	Petri et al. (2)
	Leukocytoclastic vasculitis	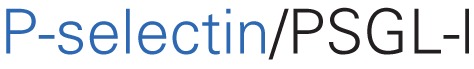	Hara et al. (3)
	Chronic cutaneous hypersensitivity	ND	Tamagawa-Mineoka et al. (4)
	Acute lung inflammation	P-selectin	Hidalgo et al. (7), Zarbock et al. (5)
Leukocyte recruitment	Glomerulonephritis	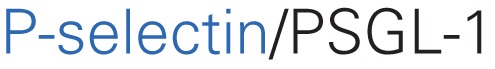	Kuligowski et al. (17)
		P-selectin	Devi et al. (19)
	Atherosclerosis	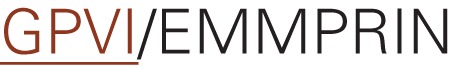	Schulz et al. (22)
		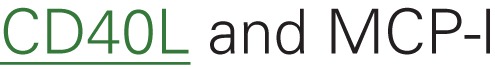	Lievens et al. (23)
		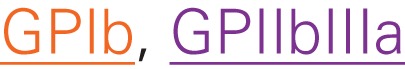	Massberg et al. (12)
		P-selectin	Huo et al. (24)
	Cerebral I/R	GPIIbIIIa	Massberg et al. (15)
		CD40/CD40L/sCD40L	Ishikawa et al. (14)
	Intestinal I/R	P-selectin	Ishikawa et al. (14)
	Abdominal sepsis	Serotonin	Duerschmied et al. (25)
		ND/Mac-1	Asaduzzaman et al. (6)
	Peritonitis	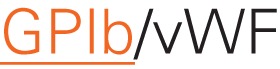	Petri et al. (2)
		Serotonin	Duerschmied et al. (25)
	Experimental colitis	CD40L	Vowinkel et al. (20)
		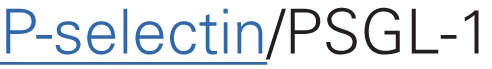	Vowinkel et al. (20)
	Aseptic wound	Serotonin	Duerschmied et al. (25)
	Acute Lung inflammation	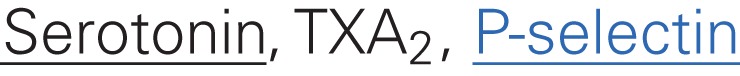	Duerschmied et al. (25), Hidalgo et al. (7), Zarbock et al. (5)
	Leukocytoclastic vasculitis	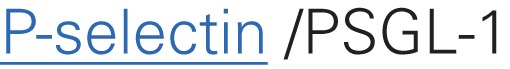	Hara et al. (3)
	Chronic cutaneous hypersensitivity	P-selectin	Tamagawa-Mineoka et al. (4)
	Allergic asthma	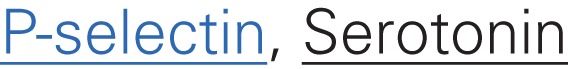	Pitchford et al. (21), Durk et al. (26)

*Examples of various inflammatory situations in which platelets were shown to regulate vascular permeability and/or leukocyte recruitment. If identified, the adhesion receptors and/or soluble factors involved are indicated. Platelet-derived factors are underlined*.

*CD40, cluster of differentiation 40, CD40L, cluster of differentiation 40 ligand, EMMPRIN, extracellular matrix metalloproteinase inducer, Mac-1, macrophage-1 antigen, MCP-1, monocyte chemoattractant protein-1, ND, non-determined, PAF, platelet-activating factor, PSGL-1, P-selectin glycoprotein ligand 1, sCD40L, soluble cluster of differentiation 40 ligand, TXA2, thromboxane A2, VEGF, vascular endothelial growth factor, vWF, von Willebrand factor*.

Remarkably, immunohistological and intravital analyses have revealed that platelet recruitment during the early phase of the inflammatory reaction mostly occurs through transient and firm adhesion of individual platelets to endothelial cells and gaps in the endothelial lining, and/or to adherent leukocytes rather than through platelet aggregation and thrombus formation (7, 9, 10, 12–14, 16, 18–20). Binding of platelets to adherent leukocytes illustrates the fact that while platelets support the recruitment of leukocytes during inflammatory reactions, the opposite is also true. Inhibition of neutrophil recruitment by immunodepletion of neutrophils or by pharmacological or genetic targeting of their adhesion receptors was indeed shown to induce a reduction in platelet recruitment in various inflammation models (7, 13, 14, 19, 27). It is also noteworthy that interactions between activated platelets and leukocytes do not only occur at the inflammatory site but also in the circulation. The signals triggered by these interactions notably induce integrin activation, thus priming leukocytes for adhesion to inflamed vessels (24, 28). Increased formation of neutrophil/platelet and/or monocyte/platelet complexes has been reported in patients with various inflammatory diseases including rheumatoid and psoriatic arthritis (29–31), allergic asthma (32), perionditis (33), ulcerative colitis (34), ischemic stroke (35), dengue infection (36), and atherosclerosis (37). Considering the contribution of platelet/leukocyte interactions to the pathogenesis of various inflammatory diseases in experimental models, in part, via promotion of neutrophil infiltration, increased platelet/leukocyte complexes in human diseases may not only reflect platelet activation but also participate in disease regulation.

The release of soluble factors by platelets is also central to their ability to modulate endothelial permeability and leukocyte infiltration. In the same way, platelets contain endothelial barrier-enhancing factors [e.g., angiopoietin-1 and sphingosine-1-phosphate (S1P)] (1), they also contain a large range of chemokines, cytokines, and other soluble mediators capable of loosening endothelial junctions and/or of activating or attracting leukocytes. Among the platelet factors that can open interendothelial junctions are factors that are not specific to platelets such as vascular endothelial growth factor (38) or platelet-activating factor (PAF) (39, 40), and serotonin, for which platelets are the major peripheral source (41). In particular, platelet serotonin was recently shown to mediate the GPVI-dependent pro-permeability effect of platelets in inflamed joints during arthritis (10) and the recruitment of neutrophils and/or eosinophils in various models of acute inflammation (25, 26).

The panel of chemokines found in platelets is extensive and includes platelet factor 4, stromal cell-derived factor-1, macrophage inflammatory protein-1α (MIP-1α), regulated upon activation, normal T cell expressed and secreted (RANTES), and thymus and activation-regulated chemokine (TARC), to name but a few (4, 24, 42). Like P-selectin, these chemokines are stored in platelet granules and their contribution to recruitment of leukocytes by platelets highlights the need for platelet activation in this process. In agreement, ADP-dependent platelet activation was required for glomerular recruitment of leukocytes by platelets (19). Moreover, while some platelet-derived soluble factors directly target endothelial cells and/or leukocytes, others like PAF, S1P, and serotonin also target platelets themselves and could therefore amplify their regulatory effects on vascular permeability and/or leukocyte recruitment. Finally, another mechanism reported for the stimulation of neutrophil recruitment by activated platelets is the up-regulation of the expression of adhesion molecules on the endothelium. Activated platelets were shown to increase the expression of ICAM-1 and αvβ3 on endothelial cells through the secretion of IL-1β (43), and to induce the secretion of Weibel–Palade bodies (44). Importantly, together, these observations that platelets can engage their adhesion receptors and release bioactive factors independently of aggregation substantiate the concept that platelet activation is a finely tuned and graduated process that allows context-dependent responses (27, 45–48).

## Platelets Regulate Neutrophil and Macrophage Effector Functions

Many studies have demonstrated the ability of platelets to regulate most of the effector functions of neutrophils and macrophages such as the production of reactive oxygen species (ROS), the secretion of neutrophil granule content, phagocytosis, or the formation of neutrophil extracellular traps (NETs). Thrombin-activated platelets were shown to stimulate the respiratory burst in unstimulated monocytes and neutrophils *in vitro* (49–51). Zalavary et al. showed that resting platelets enhanced the respiratory burst of neutrophils stimulated with IgG-opsonized yeast particles (52). However, the opposite effect was found when the chemoattractant formyl-methionyl-leucyl-phenylalanine was used as an agonist (53–56). The fact that the pro- or anti-ROS effect of platelets depends on experimental conditions such as the platelet and neutrophil agonists used again highlights how the action of platelets may ultimately be context-dependent. Several non-mutually exclusive mechanisms have been proposed to support the modulation of the oxidative burst in innate immune cells by platelets. The release of adenine nucleotide derivatives by platelets has been consistently shown to inhibit ROS generation in neutrophils (49, 53, 54). Evidence that direct cell–cell contact between platelets and monocytes/neutrophils is also important for regulation of the oxidative burst in these cells has also been provided. Prevention of platelet adhesion to neutrophils was indeed shown to suppress the modulatory effect exerted by platelets on ROS generation by neutrophils, whether it was stimulatory (50, 51, 54, 57, 58) or inhibitory (58). *In vivo* data on the actual contribution of platelets to modulation of ROS generation by innate immune cells during inflammatory reactions are, however, scarce. Nevertheless, binding of platelets to adherent neutrophils induced the generation of ROS by neutrophils in a mouse model of transfusion-related acute lung injury (7), a mechanism that might contribute to vascular damage and lung injury. Stimulation of the neutrophil respiratory burst by platelets was also proposed to contribute to proteinuria during immune-complex-induced experimental glomerulonephritis (51), as ROS were previously shown to be one of the main causes of glomerular damage in this model (59).

Several *in vitro* studies have shown that platelets can inhibit the release of myeloperoxidase and neutrophil elastase (57, 60). In contrast, platelets amplified lysozyme secretion by neutrophils stimulated with opsonized zymosan (61, 62). It is likely that as for ROS generation and endothelial permeability, platelets regulate neutrophil degranulation in a stimulus-specific manner. Some groups have demonstrated that in addition to regulating neutrophil degranulation and ROS production, platelets can also stimulate neutrophil phagocytosis by both contact-dependent and contact-independent mechanisms (52, 57). More recently, it was shown that the uptake of oxidized low-density lipoproteins (OxLDL) by monocytes was increased by direct platelet–monocyte interactions *in vitro* and *in vivo* (63). These findings partly echoed those of previous studies with the difference that the earlier studies focused on the ability of platelet secretion products to enhance macrophage uptake of OxLDL (64–66).

The production and release of cytokines by monocytes can also be regulated by platelets. Thrombin-activated platelets were shown to induce expression and secretion of MCP-1 and IL-8 by monocytes in a P-selectin-dependent manner (67). The contribution of P-selectin-mediated platelet–monocyte interactions to cytokine responses was recently investigated in patients with dengue infection. Whereas P-selectin-dependent binding of platelets from patients with dengue to monocytes from healthy volunteers induced the secretion of the IL-1β, IL-8, IL-10, and MCP-1, platelets from healthy volunteers only induced the secretion of MCP-1 (36). Modulation of cytokine levels by platelets was also reported in the cecal ligation and puncture mouse model of sepsis, a model in which deficiency in platelet GPIbα led to reduced platelet–monocyte and platelet–neutrophil interactions, and to increased circulating levels of TNF-α, MCP-1, IL-6, and MIP-1β (68). A substantial contribution of platelets to the regulation of systemic inflammatory responses was also shown in a model of pneumonia-derived sepsis (69) and in a model of thermal injury (70). In this latter model, plasma levels of TNF-α, IL-6, and MCP-1 were increased in thrombocytopenic mice, which also displayed reduced levels of TGF-β compared to control mice. In line with these studies, it was shown that secretion of PDGF by activated platelets was required for the release of MCP-1 and subsequent monocyte infiltration and killing in tissues infected with *Leishmania* parasites (71). It should be noted that thrombocytopenia led to a higher mortality rate in sepsis and thermal injury, and to reduced elimination of *Leishmania* parasites, thus demonstrating an overall beneficial role of platelets in these models. Thrombocytopenia also increased the mortality of mice infected with encephalomyocarditis virus, a situation in which platelet-TLR7-dependent formation of platelet–neutrophil aggregates played a protective role (72). These various examples where platelets exerted a beneficial effect under inflammatory conditions in some way balance those where platelets were shown to play a pathogenic role, as in glomerulonephritis (51), acute lung injury (5), atherosclerosis (12, 15), and rheumatoid arthritis (10, 73).

Neutrophil extracellular traps are extracellular webs made of DNA fibers decorated with neutrophil granule proteins that form according to a specific cell death process (74, 75). The contribution of platelets to NET formation was first demonstrated by a series of experiments showing that TLR4- and integrin-dependent platelet–neutrophil interactions led to the production of NETs capable of trapping bacteria and also causing tissue damage in models of endotoxemia and sepsis (27, 76). Stimulation of NET formation by platelets has since been shown in models of sterile acute lung injury where NETs contribute to lung endothelial damage (77, 78). Importantly, while platelets stimulate NET formation, in return, NETs cause platelet activation and aggregation, thus linking inflammation and thrombosis (79). This form of thrombosis triggered by neutrophil–platelet interactions has been called immunothrombosis and helps fight bacterial infection (80, 81). The downside of immunothrombosis is that it can also initiate deep vein thrombosis (82, 83) and contribute to cancer-associated thrombosis (84). Finally, immunothrombosis illustrates how interactions between platelets and leukocytes lead to reciprocal regulation, a notion previously introduced by earlier *in vitro* studies showing the modulation of platelet reactivity and adhesion properties by neutrophils and/or macrophages (48, 85–89).

## Platelets Prevent Inflammatory Bleeding

Inflammatory reactions have been identified as a cause of spontaneous bleeding during thrombocytopenia, thus demonstrating that platelets also intervene to prevent bleeding during inflammation. This protective role of platelets was notably established in situations where platelets were also shown to promote vascular permeability and/or leukocyte infiltration. For example, induction of thrombocytopenia resulted in immediate and severe skin bleeding during cutaneous leukocytoclastic vasculitis (90), the same model in which it caused reduced skin edema and neutrophil infiltration (3). Again, this highlights the duality of platelets that can exert apparently opposed effects during the course of a given inflammatory reaction. Nonetheless, though regulation of plasma leakage and prevention of bleeding can both be referred to as maintenance of vascular integrity, the mechanisms underlying edema formation and bleeding are essentially different. Opening of intercellular junctions favors edema formation by allowing plasma fluid and small molecules to filter out through endothelial gaps and the basement membrane. Larger elements like platelets and red blood cells are, however, retained by the vascular wall (91). For this reason, injection of the pro-permeability factor vascular endothelial growth factor (VEGF) did not cause any skin bleeding when injected in thrombocytopenic mice (92). In a similar manner, mice lacking the anti-permeability factor, S1P, in plasma exhibited basal vascular leakage of proteins and fluid in the lungs, but no bleeding (93). Therefore, while vascular permeability is tightly regulated and controlled through modulation of endothelial junctions bleeding implies vascular damage with rupture or distortion of the basement membrane mesh. There is strong evidence suggesting that neutrophils may cause this damage: in various inflammatory models, bleeding in inflamed organs of thrombocytopenic mice was prevented by depletion of neutrophils (18, 92). Prevention of inflammatory bleeding by platelets most likely relies on the inhibition or repair of neutrophil-dependent injury. Although this is unlikely to provide the full explanation, this vasculoprotective action of platelets has been shown to be independent of their ability to form thrombi. Indeed, it has been reported that platelet interactions with inflamed microvessels occur before any sign of thrombosis, and that prevention of this early initial platelet deposition results in accelerated and exacerbated local hemorrhage (18, 90). Moreover, mice lacking platelet adhesion receptors required for thrombus formation or with impaired G-protein coupled receptor (GPCR) signaling in platelets have shown no bleeding at sites of inflammation despite their compromised ability to form platelet plugs and to ensure classical hemostasis (90, 94). A recent study indicates that instead, the immunoreceptor tyrosine-based activation motif (ITAM)-associated GPVI and C-type lectin-like receptor-2 (CLEC-2) play a predominant role in the prevention of inflammatory bleeding by platelets (94, 95). Given that GPVI is the main platelet receptor for collagen, this suggests that platelets could stop bleeding by covering small areas where the basement membrane gets exposed and disrupted by recruited neutrophils. Whereas non-aggregated adherent platelets could exert a purely mechanical action by plugging small holes in the endothelial lining, recent results suggest that the release of soluble factors by platelets could further help seal vessel lesions induced by neutrophils. We have shown that even when not causing aggregation, engagement of GPVI by collagen leads to the release of soluble platelet factors capable of tightening interendothelial junctions such as angiopoietin-1 (48). Furthermore, platelets lacking the adapter protein SLP-76, which mediates GPVI-dependent platelet responses to collagen including degranulation and spreading, were shown to be unable to prevent inflammatory bleeding (94). Also, although absence of S1P is not in itself a cause of bleeding, it was shown to sensitize peripheral lymph nodes to bleeding in a mouse model of immunization characterized by high lymphocyte trafficking (95). Whether this protective role of S1P and, more specifically, of platelet S1P also applies to other inflammatory models remains to be verified but in any case, results from this study indicate that factors stimulating closure of interendothelial junctions can help stop bleeding associated with leukocyte trafficking.

The ITAM-dependent vasculoprotective action of platelets could also depend on their interactions with leukocytes. As mentioned above, platelets recruited to inflamed vessels bind to adherent leukocytes. The extracellular matrix metalloproteinase inducer (EMMPRIN) was recently identified as a counter-receptor for GPVI and is present on neutrophils and macrophages (22, 96). Additionally, podoplanin, the ligand for CLEC-2, is absent on blood endothelial cells but is expressed by inflammatory macrophages (97). ITAM-mediated interactions between platelets and innate immune cells thus represent another potential trigger for the anti-permeability and/or “neutrophil-dampening” effect of platelets.

**Figure 1:**
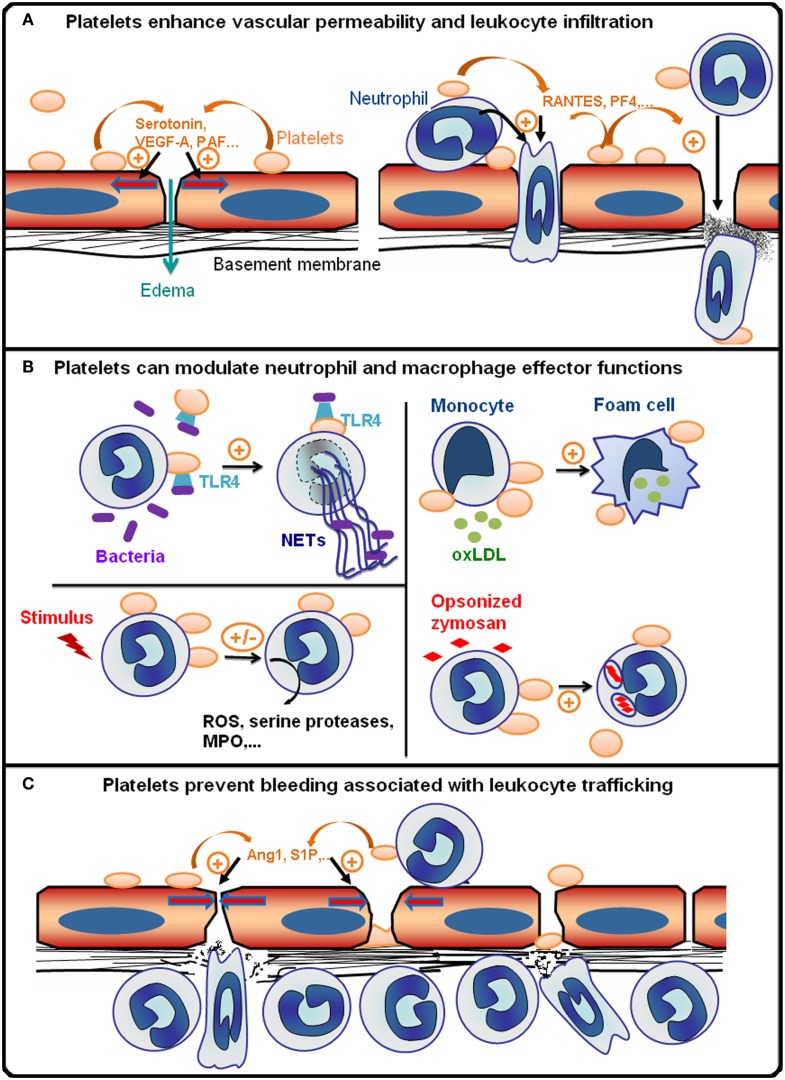
**Platelets are integral players of inflammatory reactions**. **(A)** During the early phase of inflammatory reactions, platelets are recruited to inflamed vessels and enhance vascular permeability by secreting pro-permeability factors such as serotonin, vascular endothelial growth factor A (VEGF-A), or platelet-activating factor (PAF). Platelets further enhance the recruitment and infiltration of leukocytes by secreting chemokines [e.g., activation or normal T cell expressed and secreted (RANTES) or platelet factor 4 (PF-4)], and by upregulating the expression and/or activation of adhesion molecules on leukocytes and endothelial cells via direct cell–cell contacts and secretion of pro-inflammatory cytokines. The platelet receptors (e.g., GPVI, GPIb, GPIIbIIIa, or P-selectin) supporting the interactions of platelets with inflamed vessels and/or leukocytes vary with the organ and inflammatory reactions concerned. **(B)** Platelets can modulate various leukocyte effector functions: toll-like receptor 4 (TLR4)-dependent activation of platelets promotes the formation of antibacterial neutrophil extracellular traps (NETs); platelets can also either stimulate or inhibit the production of reactive oxygen species (ROS) and/or secretion of cytokines and cytotoxic enzymes [e.g., myeloperoxidase (MPO), serine proteases] by neutrophils and macrophages depending on the inflammatory situation; platelets can stimulate phagocytosis and thus enhance foam cell formation by promoting the uptake of oxidized low-density lipoprotein by monocytes/macrophages (oxLDL). **(C)** Platelets prevent bleeding associated with leukocyte trafficking. This protective action of platelets has been shown to be independent of thrombus formation and to rely on the engagement of platelet ITAM receptors, which might cause secretion of anti-permeability factors such as angiopoietin-1 (Ang1) and sphingosine-1-phosphate (S1P) by platelets.

In conclusion, all these data showing that in a given situation, platelets can intervene to regulate vascular permeability, leukocyte infiltration, and effector functions, as well as bleeding associated with leukocyte trafficking, demonstrate that platelets are integral players of inflammatory reactions (Figure 1). Also, it appears that the pro- or anti-inflammatory character of platelets is highly dependent on parameters such as the cause of inflammation, which defines the pathways and extent of leukocyte, platelet, and endothelial cell activation, and the stage and/or endpoint readouts of the inflammatory reaction considered. In a similar manner, the overall impact of platelets on inflammatory diseases can either be beneficial or deleterious depending on the pathophysiological situation. Finally, as illustrated by the increasing interest in platelets as potential targets for the treatment of allergic inflammation (26, 98, 99), the availability of numerous anti-platelet drugs could open up new therapeutic perspectives for the many inflammatory diseases where platelets have been shown to play a pathogenic role (100).

## Conflict of Interest Statement

The authors declare that the research was conducted in the absence of any commercial or financial relationships that could be construed as a potential conflict of interest.
